# Evaluating an intervention to reduce fear of falling and associated activity restriction in elderly persons: design of a randomised controlled trial [ISRCTN43792817]

**DOI:** 10.1186/1471-2458-5-26

**Published:** 2005-03-21

**Authors:** GAR Zijlstra, JCM van Haastregt, JThM van Eijk, GIJM Kempen

**Affiliations:** 1 Universiteit Maastricht, Faculty of Health Sciences, Department of Health Care Studies, section Medical Sociology, P.O. Box 616, 6200 MD Maastricht, the Netherlands; 2 Care and Public Health Research Institute, P.O. Box 616, 6200 MD Maastricht, the Netherlands

## Abstract

**Background:**

Fear of falling and associated activity restriction is common in older persons living in the community. Adverse consequences of fear of falling and associated activity restriction, like functional decline and falls, may have a major impact on physical, mental and social functioning of these persons. This paper presents the design of a trial evaluating a cognitive behavioural group intervention to reduce fear of falling and associated activity restriction in older persons living in the community.

**Methods/design:**

A two-group randomised controlled trial was developed to evaluate the intervention. Persons 70 years of age or over and still living in the community were eligible for study if they experienced at least some fear of falling and associated activity restriction. A random community sample of elderly people was screened for eligibility; those eligible for study were measured at baseline and were subsequently allocated to the intervention or control group. Follow-up measurements were carried out directly after the intervention period, and then at six months and 12 months after the intervention. People allocated to the intervention group were invited to participate in eight weekly sessions of two hours each and a booster session. This booster session was conducted before the follow-up measurement at six months after the intervention. People allocated to the control group received no intervention as a result of this trial.

Both an effect evaluation and a process evaluation were performed. The primary outcome measures of the effect evaluation are fear of falling, avoidance of activity due to fear of falling, and daily activity. The secondary outcome measures are perceived general health, self-rated life satisfaction, activities of daily life, feelings of anxiety, symptoms of depression, social support interactions, feelings of loneliness, falls, perceived consequences of falling, and perceived risk of falling. The outcomes of the process evaluation comprise the performance of the intervention according to protocol, the attendance and adherence of participants, and the participants' and facilitators' opinion about the intervention. Data of the effect evaluation will be analysed according the intention-to-treat and on-treatment principle. Data of the process evaluation will be analysed using descriptive techniques.

## Background

Fear of falling and associated activity restriction are common in elderly persons, both in those older persons who have experienced a fall and those who have not [1,2]. Studies in older people living in the community showed that about 20 to 60 percent of these persons experience at least some fear of falling [2-6] and about 20 to 55 percent report activity restriction due to fear of falling [1,5,7-9]. In this paper, we focus on the design of a randomised controlled trial evaluating a cognitive behavioural group intervention, which aims to reduce fear of falling and associated activity restriction in elderly persons.

In one of the first reported studies on fear of falling, Vellas and colleagues indicated that fear of falling may lead to a debilitating spiral marked by loss of confidence and reduced activity, resulting ultimately in a loss of independence [10]. In later studies this observation was strengthened. Fear of falling was found to be associated with several adverse factors, including decreased quality of life [3,11], decreased mobility [3,11], functional decline [3,11,12], falls [13], and institutionalisation [11]. These factors may not only have an adverse influence on the physical health status of elderly persons, but on the social and mental health status as well. Therefore, reducing fear of falling and associated activity restriction in older persons may improve their health status. However, until now only a few interventions have been developed and evaluated specifically to reduce fear of falling in elderly people living in the community [14-16].

In our trial we will evaluate one of these interventions in the Netherlands, a cognitive behavioural group intervention called A Matter of Balance (AMB). This intervention has originally been developed and evaluated in the US and aims to reduce fear of falling and associated activity restriction in elderly persons [15]. The intention-to-treat analysis showed statistically significant effects for increased activity (mobility and intended activity) directly after the intervention. The on-treatment analysis (including participants who attended five or more out of eight sessions) showed statistically significant improvement in falls efficacy, perceived ability to manage falls, and activity (mobility) directly after the intervention. At 12 months, this latter group showed statistically significant improvement in falls efficacy and activity (mobility range and social function).

Based on the reported effectiveness of AMB in the US and the aspiration to implement AMB in the Dutch health care system, we decided to perform a trial to assess its effectiveness in the Netherlands. For this purpose, the intervention protocol of AMB was translated and adapted for the Dutch setting (AMB-NL) (Zijlstra et al., development of intervention protocol, submitted). The current paper presents the design of a randomised controlled trial evaluating AMB-NL in Dutch older persons living in the community.

### Aims

The primary aim of the effect evaluation was to study the effects of AMB-NL on fear of falling, avoidance of activity due to fear of falling, and daily activity in older persons living in the community in the Netherlands. The secondary aim was to study the effects of this intervention on perceived general health, self-rated life satisfaction, activities of daily life, feelings of anxiety, symptoms of depression, social support interactions, feelings of loneliness, falls, perceived consequences of falling, and perceived risk of falling. The aim of the process evaluation was to gain insight into factors potentially influencing the effectiveness of the intervention and factors facilitating future implementation of AMB-NL in the Dutch health care setting, if the intervention proves to be effective.

## Methods/design

### Study design

**Figure 1:**
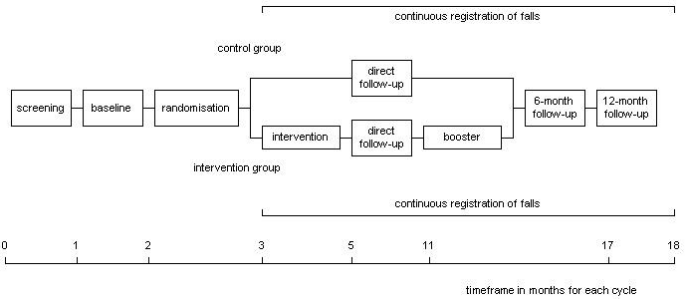
Study design

A two-group randomised controlled trial with participants being randomly allocated to either an intervention or a control group has been developed to evaluate AMB-NL. Selecting potential participants, conducting the intervention, and collecting data were performed in five consecutive cycles. The first cycle started in November 2002 and the last cycle in July 2003. Each cycle lasted about 18 months and included respectively: screening for eligible participants, baseline measurement, randomisation (allocation to the intervention or control group), the intervention period, a follow-up measurement directly after the intervention period, a booster session at six months after the intervention, a follow-up at six months after the intervention (directly after the booster session), and a final follow-up at 12 months after the intervention. The Medical Ethics Committee of the Maastricht University/Academic Hospital Maastricht granted approval for conducting this trial. The study design is presented in figure 1.

### Settings and locations

Two communities, Heerlen and Maastricht, situated in the southeastern part of the Netherlands were selected for participation in the trial. As screening for potential participants and conducting the intervention was performed in five cycles, both communities were divided into five sections proportional to the number of potential participants in contiguous neighbourhoods. Intervention sessions were conducted in local community centres or homes for the elderly preferably located in the centre of those neighbourhoods. Transportation to the intervention location was offered to participants who expected difficulties to reach the location, for example due to health problems.

### Participants

Elderly persons were eligible for study if they met all of the following criteria: 1) reporting at least some fear of falling; 2) reporting at least some associated avoidance of activity; 3) living in the community; and 4) being 70 years of age or over. Elderly persons confined to bed, restricted by permanent use of wheelchair, or waiting for nursing home admission were excluded for study.

Immediately before screening for eligible participants in each of the five cycles, addresses of older people living in the community who were 70 years of age or over at 1 January 2003 were randomly selected by municipal registry offices. Selected persons were sent information about the trial and a brief self-administered screening questionnaire. This questionnaire assessed socio-demographic and fall-related variables, all inclusion and exclusion criteria, and willingness to participate. Persons interested in participating in the trial were asked to sign an informed consent form enclosed in the questionnaire. All selected persons were requested to complete and return the questionnaire, even if they lacked interest in participating in the trial. A postage free envelope was enclosed for returning the questionnaire. If the questionnaire was not returned in a fortnight, a reminder letter to return the questionnaire was sent. Those persons who signed the informed consent form, were willing to participate, and met all other eligibility criteria were invited to participate in the study.

### Randomisation

Randomisation was carried out directly after baseline measurement and was performed per community to ensure having both an intervention group and a control group in each of the two communities. During each cycle two intervention groups were composed per community. Per cycle, approximately half of the participants were allocated to the intervention group (with a maximum of 15 participants per intervention group). Participants allocated to the intervention group were then randomly allocated to one of the two groups in their own community. Participants allocated to the control group received no intervention as a result of this trial. An independent researcher conducted randomisation by selecting random samples using SPSS12.0 for Windows.

### Intervention

The intervention AMB-NL is a translated and adapted version (Zijlstra, et al., development of intervention protocol, submitted) of a cognitive behavioural group intervention for older persons living in the community developed by Tennstedt and colleagues [15]. This intervention has been developed to reduce fear of falling and promote physical, social, and functional activity in elderly persons living in the community. Principles of cognitive restructuring [17] are applied by focusing on changing attitudes and self-efficacy beliefs with respect to falling before attempting to change actual behaviour. To attain a reduction in fear of falling, the intervention aims to increase self-efficacy beliefs with regard to falling as well as the sense of control over falling. Four strategies are used to accomplish these aims: (1) restructuring misconceptions to promote a view of fall risk and fear of falling as controllable; (2) setting realistic goals for increasing activity; (3) changing the environment to reduce fall risk; and (4) promoting physical exercise to increase strength and balance [15].

The intervention consists of eight weekly group sessions lasting two hours. Six months after the eighth session a booster session is scheduled. The main topics in each of the sessions of AMB-NL, presented in table 1, were discussed similarly: (1) introduction; (2) participant's point of view; (3) positive and negative aspects concerning the topic; (4) association with falls or fear of falling; and (5) implementation in the participant's daily life. A more extensive description of the intervention will be published elsewhere (Zijlstra et al., development of intervention protocol, submitted).

**Table 1:** Main contents of the Dutch version of AMB (AMB-NL)

Session contents of AMB-NL

Session 1: Introduction to the Program

Starting a group intervention (e.g. getting acquainted)
Background information on fear of falling (e.g. incidence, impact)
Beliefs and disbeliefs about fear of falling
Shifting from negative to positive thinking patterns
Finding personal solutions to deal with fear of falling

Session 2: Exploring Thoughts and Concerns about Falling

Attitudes related to fear of falling and challenging them
Adaptive responses to counter misconceptions about falls
Unhelpful thoughts and their effects regarding to feelings and behaviour
Shifting from self-defeating to self-motivating thoughts

Session 3: Exercise and Fall Prevention

Misconceptions regarding physical exercise for elderly people
Potential consequences of inactivity and benefits of physical activity
Staying or becoming physically active to prevent falls
Recognising and overcoming barriers to stay or become physically active
Appropriate physical exercises for elderly people and fall prevention
Practicing simple physical exercises

Session 4: Assertiveness and Fall Prevention

Association between assertiveness and fall prevention
Potential benefits of being assertive
Reducing fall risk by being assertive in a proper fashion
Addressing physical risk factors for falls
The influence of physical exercise on physical characteristics (e.g. blood pressure)
Practicing physical exercises

Session 5: Managing Concerns about Falling

Developing and implementing a personal physical exercise program
Shifting from self-defeating to self-motivating thoughts regarding physical activity and fall risk
Practicing physical exercises
Midcourse evaluation to review all main topics

Session 6: Recognising Fall-ty Habits

Identifying and managing risk-taking behaviour in daily life
Prioritising fall risk behaviours
Searching for suitable, personal solutions to change risk-taking behaviour into safe actions
Planning behaviour change strategies
Setting goals for activities one would like to carry out
Shifting from negative thoughts associated with planned activities to positive responses
Practicing physical exercises
Discussing falls and seeking help after a fall

Session 7: Recognising Fall Hazards in the Home and Community

Potential fall hazards in homes and community
Recognising and eliminating environmental hazards by finding simple solutions
Discussing displayed assistive devices which improve safety
Practicing physical exercises

Session 8: Practicing No Fall-ty Habits

Practicing assertiveness skills for locating and utilising resources to prevent falls
Understanding that risk-taking behaviour can be eliminated
Practicing physical exercises

Booster session

Discussing personal experiences with falls and fear of falling
Shifting from self-defeating to self-motivating thoughts
Exercise and fall prevention
Potential fall hazards in homes and community
Change risk-taking behaviour into safe actions
Practicing physical exercises

Nurses qualified in the field of geriatrics and working for home care agencies were trained as facilitators of the intervention. Except for the first session when two facilitators were present, each intervention session was conducted by one facilitator. Monthly meetings with facilitators and researchers were scheduled to evaluate and discuss the progress of the trial, the intervention and associated matters. Participants were informed to notify the facilitators or researchers if they were unable to attend a session. After the session, facilitators contacted those participants who were absent and briefly discussed the topics of that session. However, those participants who were absent in all of the first three sessions were excluded from further participation in the intervention due to an unbridgeable deficiency in knowledge.

### Measures

#### Effect evaluation

##### Primary outcome variables

The primary outcomes of the effect evaluation are fear of falling, avoidance of activity due to fear of falling, and daily activity. *Fear of falling *was assessed by three different measures. First, respondents indicated the frequency of fear of falling when asked "Are you afraid of falling?" (1 = never to 5 = very often). Second, on the 10-item Falls Efficacy Scale (FES) respondents indicated how worried they are about falling while carrying out several indoor activities of daily living (1 = not at all worried to 4 = very worried) [7,18]. Four items about outdoor activities [19] were added. Finally, a four-item scale was used to assess the respondent's perceived control over falling (PCOF) [20]. This scale focuses on the perceived control over the environment and one's own mobility and ability to do things to prevent falls and reduce fear of falling. The frequency of *avoidance of activity *due to fear of falling was assessed by the question "Do you avoid certain activities due to fear of falling?" (1 = never to 5 = always). *Daily activity *was assessed by the Frenchay Activities Index (FAI) [21,22]. The FAI measures the frequency in which daily activities that reflect the broader everyday activities of normal living are performed [21]. An overview of the primary and secondary outcomes measured during the course of the study is presented in table 2.

**Table 2:** Primary and secondary outcome measures of the effect evaluation

Variables	No. of items	Range*		S		B		FU1		FU2		FU3
*Primary outcome measures*												
fear of falling	1	1 to 5		SQ		Q		Q		Q		Q
fall-related self-efficacy (FES) [7]	10	10 to 40		-		TI		TI		TI		TI
outdoor items fall-related self-efficacy [19]	4	4 to 16		-		TI		TI		TI		TI
perceived control over falling (PCOF) [20]	4	4 to 20		-		Q		Q		Q		Q
avoidance of activity due to fear of falling	1	1 to 5		SQ		Q		Q		Q		Q
daily activity (FAI) [21, 22]	15	15 to 60		-		Q		Q		Q		Q
*Secondary outcome measures*												
perceived general health (MOS SF-20 item one) [23, 24]	1	1 to 5		SQ		Q		Q		Q		Q
self-rated life satisfaction [25]	1	1 to 7		-		Q		Q		Q		Q
activities of daily life (ADL subscale of the GARS) [26]	11	11 to 44		-		TI		TI		TI		TI
feelings of anxiety (HADS) [27, 28]	7	0 to 21		-		Q		Q		Q		Q
symptoms of depression (HADS) [27, 28]	7	0 to 21		-		Q		Q		Q		Q
social support interactions (SLL12-I) [29]	12	12 to 48		-		Q		Q		Q		Q
feelings of loneliness	1	1 to 6		-		Q		Q		Q		Q
number of falls in the previous 6 months	1	1 to 6		SQ		-		-		Q		Q
number of falls in the previous 2 months	1	1 to 6		-		Q		Q		-		-
number of indoor falls	1	N/A		-		C>		C>		C>		C>
number of outdoor falls	1	N/A		-		C>		C>		C>		C>
number of times medical attention required due to falls	1	N/A		-		C>		C>		C>		C>
perceived consequences of falling (CoF) [30]	12	12 to 48		-		TI		TI		TI		TI
perceived risk of falling (RoF) [30]	3	3 to 12		-		TI		TI		TI		TI

* The underlined scores indicate the most favourable scores.

S = screening; B = baseline; FU1 = direct follow-up; FU2 = 6-month follow-up; FU3 = 12-month follow-up

SQ = screening questionnaire; Q = questionnaire; TI = telephone interview; C> = calendar (continuous registration)

N/A = not applicable

##### Secondary outcome variables

The secondary outcomes that were assessed are: *perceived general health *(item one of the MOS SF-20) [23,24], *self-rated life satisfaction *(seven point satisfaction rating) [25], *activities of daily life *(ADL subscale of the Groningen Activity Restriction Scale (GARS)) [26], *feelings of anxiety *(Hospital Anxiety and Depression Scale (HADS)) [27,28], *symptoms of depression *(HADS) [27,28], *social support interactions *(SSL12-I) [29], *feelings of loneliness *(6 point Likert scale), *falls*, *perceived consequences of falling *(CoF) [30], and *perceived risk of falling *(RoF) [30]. *Feelings of loneliness *were assessed by the question 'During the past four weeks, how often did you feel lonely?' (1 = all the time to 6 = never). *Falls *were registered by both a one-item question and a fall calendar. The one-item question assessed how frequently the participant had fallen during the past few months (1 = never to 6 = 5 or more falls). The fall calendar was used for continuous registration of falls during the course of the trial. If a fall occurred, participants indicated on the calendar: (a) the location of the fall (indoor or outdoor); and (b) the number of times medical care was received due to the fall.

##### Additional variables

Several additional variables were assessed to provide insight into the population, to interpret the outcomes of the trial, and to study the underlying mechanisms of the intervention. *Socio-demographic variables *assessed during the process of screening were: age, gender, marital status, living condition, living alone or not, and educational level. *Health-related variables *assessed during the telephone interview of the baseline measurement were: chronic medical conditions (a 19-item checklist) [31], cognitive status (modified version of the Telephone Interview for Cognitive Status (TICS)) [32], and impaired vision and hearing (a four-item questionnaire) [33]. Other health-related variables assessed at baseline and follow-up telephone interviews were: use of healthcare (for example, number of visits to the general practitioner) and use of assistive devices. *Additional data *assessed with baseline and follow-up questionnaires were: general self-efficacy (GSE) [34,35], physical self-efficacy (PSE) [36], social self-efficacy (SSE) [34], and mastery [37]. An overview of the additional health-related scales and self-efficacy and mastery scales is presented in table 3.

**Table 3:** Additional measures during the trial

Description of the variables	No. of items	Range*		B		FU1		FU2		FU3
chronic medical conditions [31]	19	0 to 19		TI		-		-		-
cognitive status (TICS) [32]	25	0 to 41		TI		-		-		-
impaired vision and hearing [33]	4	4 to 16		TI		-		-		-
use of health care	6	N/A		TI		TI		TI		TI
use of assistive devices	14	N/A		TI		TI		TI		TI
general self-efficacy (GSE) [34, 35]	16	16 to 80		Q		Q		Q		Q
physical self-efficacy (PSE) [36]	10	10 to 50		Q		Q		Q		Q
social self-efficacy (SSE) [34]	6	6 to 30		Q		Q		Q		Q
mastery [37]	7	7 to 35		Q		Q		Q		Q

* The underlined scores indicate the most favourable scores.

B = baseline; FU1 = direct follow-up; FU2 = 6-month follow-up; FU3 = 12-month follow-up

Q = questionnaire; TI = telephone interview

N/A = not applicable

#### Process evaluation

The process evaluation was aimed at gaining insight into factors potentially influencing the effectiveness of the intervention and factors facilitating future implementation of the intervention. Four main outcome measures were identified: (1) performance of the intervention according to protocol; (2) attendance of participants; (3) adherence of participants; and (4) opinion of participants and facilitators about the intervention. Table 4 provides an overview of the outcomes of the process evaluation during the course of the trial.

**Table 4:** Outcome measures of the process evaluation

Variables	BDI	FU1	FU2	FU3
*Performance intervention according to protocol*				
duration of the sessions	R^f^	-	-	-
deviations from protocol	R^f^	-	-	-
*Attendance of participants*				
reasons for refusal before the start of the intervention	TI^p^	-	-	-
number of sessions visited by each subject	R^f^	-	R^f^	-
reasons for stopping during the intervention period	TI^p^	-	TI^p^	-
*Adherence of participants*				
adherence to homework assignments	-	Q^p^/Q^f^	-	-
adherence to physical exercise	-	Q^p^/Q^f^	Q^p^	Q^p^
*Opinion about intervention*				
overall judgement about the intervention	-	Q^p^/Q^f^	-	-
judgement about the facilitators	-	Q^p^/Q^f^	-	-
benefit experienced by participants	-	Q^p^/Q^f^	Q^p^	Q^p^
strong and weak aspects of the intervention	-	Q^p^/Q^f^	-	-
recommendations for improvement	-	Q^p^/Q^f^	Q^f^	-

BDI = before or during intervention; FU1 = direct follow-up; FU2 = 6-month follow-up; FU3 = 12-month follow-up

R = registration form filled in after each session; Q = questionnaire; TI = telephone interview

Data collected from: ^f ^= facilitator; ^p ^= participant

### Data collection

Data for the effect evaluation were gathered by means of self-administered questionnaires, fall calendars, and telephone interviews. Trained interviewers, who were blinded for group allocation, conducted the interviews. After baseline measurement participants received a fall calendar; every three months a sheet of the calendar was to be returned to the research team. Data for the process evaluation collected from the participants were completed by means of self-administered questionnaires and short telephone interviews. Registration forms and self-administered questionnaires were used to gather data from the facilitators. These data were discussed and illustrated by the facilitators in two evaluative meetings.

As recommended by Hollis and Campbell [38], non-compliant participants of the intervention group were approached for all follow-up measurements and participants with missing data were contacted to ensure completion of data. At five and 11 months after the intervention, newsletters were sent to keep the participants interested in participating in the trial and to notify them about its progress.

### Sample size and power

Sample size calculations were based on experiences in evaluative studies among older persons in the Netherlands [39] and the US [15] where fall-related self-efficacy [7] was assessed. To detect a mean difference of at least 2.5 points on fall-related self-efficacy between the intervention and control group (SD is 6.0; equivalent to an effect size of .42), at least 117 participants in each group were necessary to meet a power of 80 percent at alpha .01 (one-sided). Taking a dropout rate of 30 percent during the trial into account, a total number of 180 participants per group (n = 360) were needed to enrol in the trial. Based on unpublished data of Van Haastregt and colleagues (study described in [39]), we estimated that approximately 14.5 percent of the older persons who returned the screening questionnaire would meet all inclusion criteria and exclusion criteria and would be interested in participating in the trial. With an estimated response rate of 55 percent, a minimum sample of 4500 older persons aged 70 or over needed to be approached with a screening questionnaire.

### Analysis

Descriptive techniques will be used to describe participants of the trial. To detect differences between intervention and control group, baseline variables will be compared using the appropriate tests. Baseline variables of compliant and non-compliant participants of the intervention group will be compared as well. Compliant participants are participants who attended five or more sessions [15], not including the booster session.

Data of the effect evaluation will be analysed according the intention-to-treat and on-treatment principle. Univariate and multivariate techniques will be applied to examine the differences in the intervention and control group with regard to the primary and secondary outcome measures at the follow-up measurements. Effect sizes [40] will be calculated to quantify the size of the difference between both groups. Subgroup analysis will be performed with several potential effect modifiers, like cognitive status or educational level.

Data of the process evaluation will be analysed using descriptive techniques.

## Discussion

### Progress of the study

A random selection of older persons aged 70 or over living in the community was screened for eligibility in five cycles between November 2002 and July 2003. A total of 7431 older persons rightfully received the screening questionnaire. The response rate was 58.9 percent. During the course of the study the number of people who were sent a screening questionnaire was increased based on two grounds. First, in contrast to our estimate of 14.5 percent, only about 10 percent of the participants in the first cycle met our eligibility criteria and were willing to participate. Second, about 25 percent of the persons participating in the baseline measurement dropped out before randomisation. In total, 540 participants were included in the trial; 260 participants were allocated to the intervention group and 280 to the control group. The data collection was completed in February 2005. Currently, preparations for the analyses of the data of the screening and process evaluation are being made. Data of the effect evaluation will be available in 2005.

### Future implementation

Implementation in the Dutch setting has been taken into account throughout the development of AMB-NL (Zijlstra et al., development of intervention protocol, submitted). If the results of the trial show the effectiveness of AMB-NL, recommendations will be developed to implement the intervention in the Dutch health care setting and a manual of the intervention, updated with the experiences of the trial, will be made available.

## Abbreviations

AMB – A Matter of Balance

AMB-NL – the Dutch version of A Matter of Balance

## Competing interests

The author(s) declare that they have no competing interests.

## Authors' contributions

All authors contributed to the development of the design of this study. G.A.R. Zijlstra drafted the manuscript with input from the other authors. All authors read and approved the final manuscript.

## Pre-publication history

The pre-publication history for this paper can be accessed here:


